# Impact of the COVID-19 Aerosol Box on the Intubation Success Rate

**DOI:** 10.7759/cureus.94672

**Published:** 2025-10-15

**Authors:** Nurul Najwa Mohd Noor, Maryam Budiman, Rufinah Teo, Shuhaida Che Shaffi, Nadia Md Nor

**Affiliations:** 1 Department of Anaesthesiology and Intensive Care, Hospital Sultan Abdul Aziz Shah, Universiti Putra Malaysia, Serdang, MYS; 2 Department of Anaesthesiology and Intensive Care, Hospital Canselor Tuanku Muhriz, Universiti Kebangsaan Malaysia, Kuala Lumpur, MYS; 3 Department of Anaesthesiology and Critical Care, Hospital Seberang Jaya, Pulau Pinang, MYS

**Keywords:** aerosol box, covid-19, intubation difficulty, intubation difficulty score, time to tracheal intubation

## Abstract

Introduction

The aerosol box was introduced as a protective barrier to reduce healthcare workers' exposure to aerosols during intubation. However, concerns remain regarding its impact on intubation efficiency and patient safety. This study compares intubation success rate, time to tracheal intubation, and intubation difficulty with and without the aerosol box.

Methods

A randomized controlled trial was conducted on 60 adult patients (ASA I-II, BMI ≤35 kg/m²) undergoing elective surgery under general anesthesia. Participants were randomized into two groups: intubation with an aerosol box (Group A) and intubation without an aerosol box (Group B). Seven experienced anesthesia trainees performed the intubations. The primary outcome was time to successful tracheal intubation. Secondary outcomes included first-pass intubation success, Intubation Difficulty Score (IDS), and issues related to aerosol box usage.

Results

There were no statistically significant differences in demographic characteristics between the groups. All patients had successful first-pass intubation. The mean intubation time was significantly longer in Group A compared to Group B (30.73 ± 2.9 vs. 23.40 ± 2.0 seconds, p < 0.01). There was no significant difference in intubation difficulty between groups. Common issues encountered with the aerosol box included migration off the bed in five cases (17.2%) and the need for external assistance in five cases (17.2%). The learning curve was comparable among participants.

Conclusion

Intubation with or without an aerosol box resulted in a 100% first-pass success rate with no significant difference in intubation difficulty. However, the aerosol box significantly increased intubation time.

## Introduction

In late 2019, the world witnessed the emergence of a novel coronavirus, later named severe acute respiratory syndrome Coronavirus 2 (SARS-CoV-2), which led to a global outbreak originating in Wuhan, China. Initially referred to as “2019 novel coronavirus” (2019-nCoV), the World Health Organization officially named the disease COVID-19 (coronavirus disease 2019) in February 2020. By March 2020, COVID-19 was declared a pandemic by the WHO, signifying a severe global health crisis. In Malaysia, the first case was detected on January 24, 2020, and as of June 25, 2020, there were 8,600 confirmed cases with a 1.4% fatality rate [1]. 

Similar to other coronaviruses, such as Middle East respiratory syndrome coronavirus (MERS-CoV) and SARS, SARS-CoV-2 is highly contagious and primarily transmitted through respiratory droplets and direct contact with contaminated surfaces. Procedures involving airway management, such as manual ventilation, airway suctioning, and intubation, are classified as aerosol-generating procedures (AGPs), posing significant risks of viral transmission to healthcare workers [2,3]. Studies have demonstrated that upper airway secretions contain the highest viral load, further emphasizing the heightened risk to those performing these procedures [4]. 

During the early days of the COVID-19 pandemic, the intubation barrier box, also known as the aerosol box, was introduced to mitigate viral spread during airway procedures. This transparent, plastic cube was designed by Dr. Lai Hsien Yung from Taiwan and aimed to protect healthcare workers by enclosing the patient’s head during intubation [5,6]. The device features two circular ports through which clinicians can insert their arms to perform the procedure, potentially limiting aerosol spread. The creation of this device was driven by the critical shortage of personal protective equipment (PPE) during the early stages of the pandemic.

However, concerns have emerged regarding the safety of the aerosol box, particularly its impact on the efficacy of intubation, as delays or complications during intubation can increase the risk of hypoxia for patients. While research on the aerosol box remains limited, one notable study published in May 2020 examined the use of two different versions of the device in a simulation environment. The study found that the use of the aerosol box significantly prolonged intubation times and lowered first-pass success rates, even among experienced airway specialists [7]. Despite the lack of extensive clinical evidence on its safety and efficacy, aerosol boxes were widely adopted in various healthcare settings for tracheal intubation of COVID-19 cases, including our own.

This study aimed to assess the intubation success rate with and without the use of the aerosol box in clinical practice. Secondary objectives included comparing the time to tracheal intubation and evaluating the ease of intubation between the two groups.

## Materials and methods

This prospective, randomized controlled study was approved by the Research Committee of the Department of Anesthesiology and Intensive Care, Universiti Kebangsaan Malaysia Medical Centre (UKMMC), and was registered in ClinicalTrials.gov (NCT06042829), as well as the Medical Research and Ethics Committee of UKMMC, under the research code HTM-2020-006. The study was conducted in the General Operating Theatre (GOT) at UKMMC over a period spanning from December 2020 to November 2021.

Seven anesthesiology trainees participated in this study, each possessing a minimum of five years of experience in anesthesia and prior exposure to intubation using an aerosol box. Before the commencement of the study, all trainees underwent a standardized refresher session in which they practiced intubation using the aerosol box on a manikin. Following the training, each trainee was required to complete ten successful intubations on a manikin and ten successful intubations on patients to ensure proficiency and standardization of technique. During the intubation process, all trainees wore a three-ply surgical mask and disposable gloves as part of their protective equipment.

Written informed consent was obtained from all patients enrolled in the study. The inclusion criteria consisted of adult patients classified as American Society of Anesthesiologists (ASA) Physical Status I or II, who were scheduled for elective surgery under general anesthesia. Patients were excluded if they had a history of claustrophobia, a body mass index (BMI) exceeding 35 kg/m², or anatomical features suggestive of a difficult airway, such as a receding chin, intraoral or large neck masses, limited neck extension, or a small mouth opening. All recruited patients were reviewed in the ward a day prior to surgery, during which their demographic data, including age, gender, race, weight, height, BMI, and ASA physical status, were recorded. Airway assessments, including the Mallampati score and thyromental distance, were also conducted and documented. Each patient fasted for at least six hours before the operation and received oral midazolam as premedication before being transferred to the operating theatre.

Randomization of patients into two groups was conducted using a computer-generated randomization process. Patients assigned to Group A underwent tracheal intubation with the use of an aerosol box, whereas those in Group B underwent tracheal intubation without the aerosol box.

**Figure 1:**
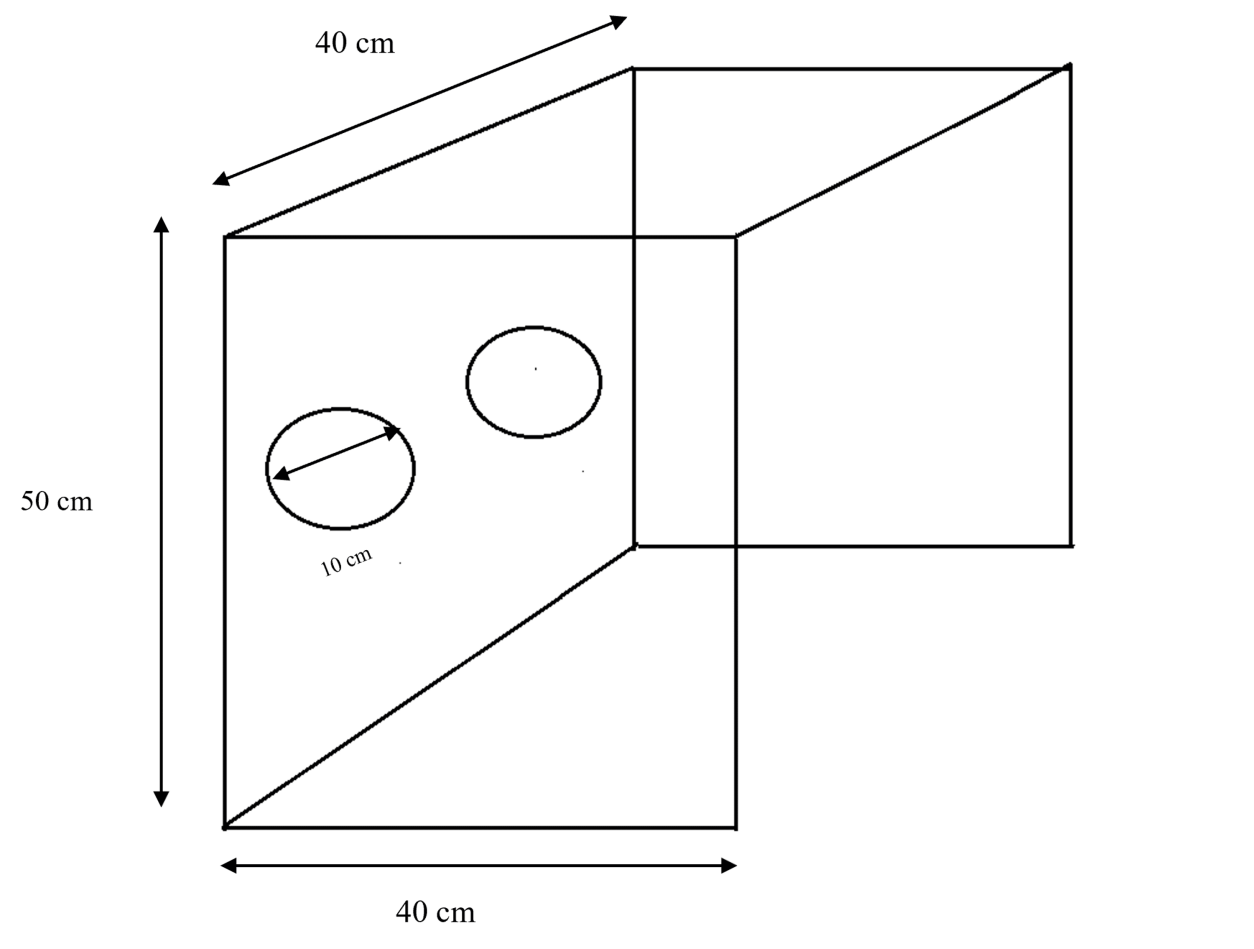
Aerosol box

On the day of surgery, intravenous access was established for all patients, and an infusion of 0.9% normal saline was initiated. Standard monitoring, including continuous electrocardiography (ECG), non-invasive blood pressure monitoring, pulse oximetry, and capnography, was applied. Each patient was positioned in the supine position with the head placed in a neutral position and stabilized using a doughnut gel pad. The aerosol box used in this study was identical to the original version designed by Dr. Lai Hsien Yung [5]. The device, as shown in Figure 1, consisted of a transparent plastic cube with dimensions of 40 cm in height, 40 cm in width, and 50 cm in depth. It had two circular arm holes measuring 10 cm in diameter, through which the anesthesiology trainee passed their arms to perform intubation. In patients randomized to Group A, the aerosol box was placed over the patient’s head and shoulders, and the height of the operating table was adjusted to align with the trainee’s umbilical level.

All patients underwent three minutes of preoxygenation before the induction of anesthesia. Anesthesia was induced using intravenous fentanyl at a dose of 2 mcg/kg, intravenous propofol at a dose of 2 to 2.5 mg/kg, and intravenous rocuronium at a dose of 0.6 mg/kg. After adequate neuromuscular relaxation was confirmed, intubation was performed using a C-MAC® video laryngoscope (KARL STORZ Tuttlingen, Germany) with a cuffed endotracheal tube. The choice of laryngoscope blade size and endotracheal tube size was determined by the anesthesiology trainee and documented accordingly. A stylet was used to pre-shape the endotracheal tube to match the curvature of the C-MAC® blade (KARL STORZ Tuttlingen, Germany). Successful intubation was confirmed through the presence of capnography tracing and bilateral lung auscultation.

A maximum of three intubation attempts was permitted in this study. If a patient could not be intubated successfully after three attempts, the case was classified as a failed intubation and recorded as such. In the event of a failed intubation, subsequent airway management was conducted in accordance with the Difficult Airway Society (DAS) 2015 guidelines for the management of unanticipated difficult tracheal intubation in adults.

The primary outcome of this study was the intubation success rate, which was defined as the proportion of successful intubations achieved within the maximum of three permitted attempts. Secondary outcomes included the time to tracheal intubation, the intubation difficulty score, and subjective assessments of difficulty encountered by the anesthesiology trainees. The time to tracheal intubation was defined as the interval from the insertion of the laryngoscope blade past the front incisors until the first detectable upstroke of the capnography tracing. This time was recorded using a dedicated stopwatch by an assigned assistant. The degree of intubation difficulty was assessed using the Intubation Difficulty Scale (IDS), which is a composite numerical score based on seven parameters associated with difficult intubation [7]. These parameters included the number of supplementary attempts, the number of supplementary operators required, the use of alternative intubation techniques, the grade of glottic exposure according to the Cormack-Lehane classification, the lifting force applied during laryngoscopy, the application of external laryngeal pressure, and the position of the vocal cords. Based on the total IDS score, intubation difficulty was categorized into three levels: an IDS score of 0 was classified as an easy intubation, a score between 1 and 5 indicated a slightly difficult intubation, and a score of 6 or greater signified moderate to major difficulty.

In addition to these quantitative outcomes, anesthesiology trainees were surveyed on their subjective experience of using the aerosol box. They were asked four yes-or-no questions assessing whether they encountered arm discomfort while using the box, whether there was contact between the laryngoscope and the box, whether the aerosol box migrated off the bed during intubation, and whether additional assistance was required to hold the box in place. If the aerosol box needed to be removed at any point during the intubation process, the case was considered a dropout, and the reason for removal was documented.

The required sample size was determined using the Snedecor and Cochran formula, based on data from a previous study by Begley et al. and Snedecor and Cochran [8,9]. A total of 60 subjects was required to achieve sufficient statistical power, with an additional 20% accounted for to compensate for potential dropouts. Statistical analysis was performed using IBM SPSS Statistics for Windows, Version 25 (Released 2017; IBM Corp., Armonk, New York, United States). Data were expressed as mean ± standard deviation, median with interquartile range, or frequency with percentage, as appropriate. Between-group comparisons were conducted using the independent t-test, while skewed data were analyzed using the Mann-Whitney U test. Categorical variables were compared using the chi-square test or Fisher’s exact test when necessary. A p-value of less than 0.05 was considered statistically significant. 

## Results

**Figure 2:**
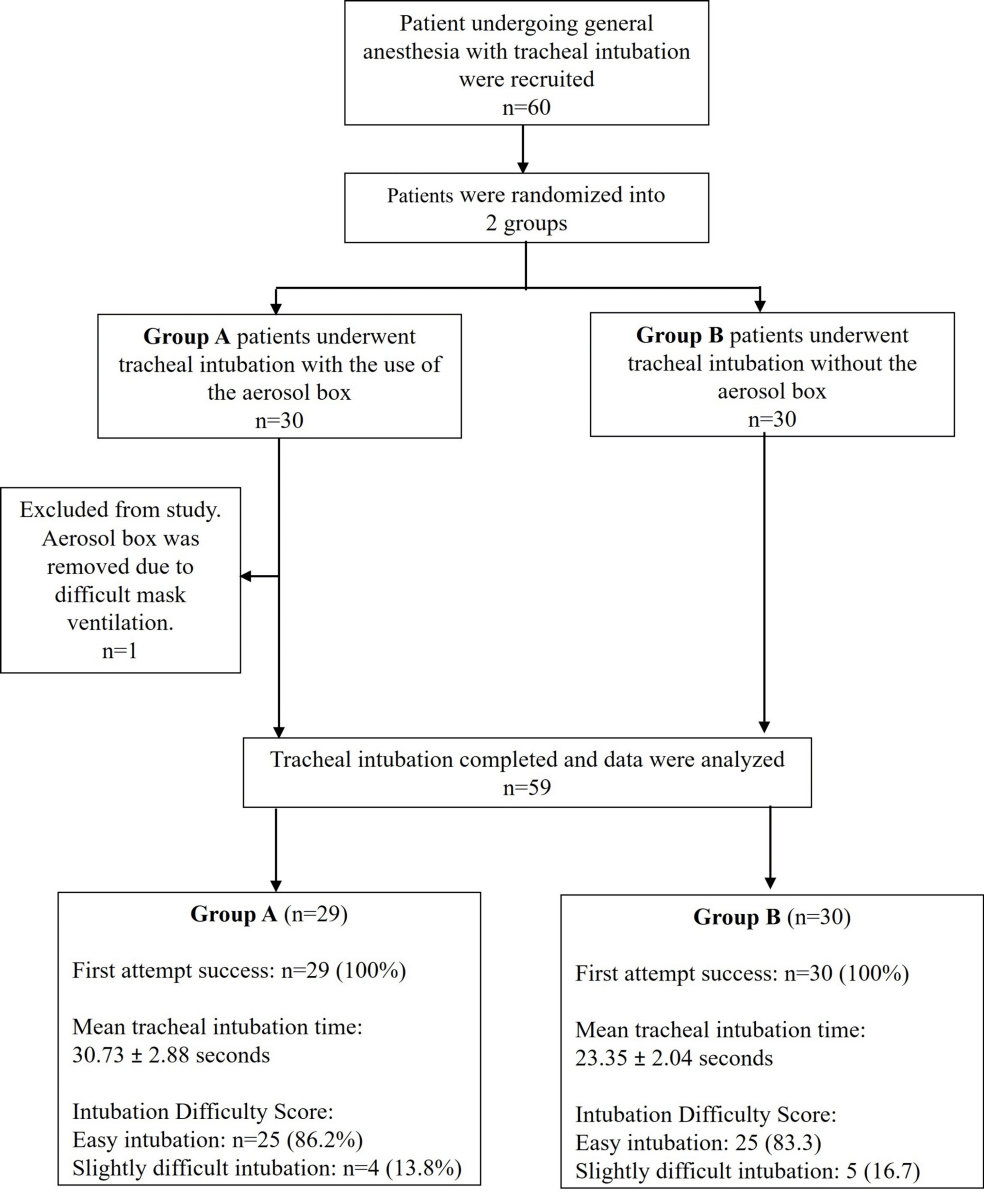
CONSORT diagram of the study CONSORT: Consolidated Standards of Reporting Trials

A total of 60 patients were recruited, and all patients had a Mallampati score of I and II, with a thyromental distance of more than three finger breadths. A total of 29 patients were randomized into Group A, where tracheal intubation was performed using an aerosol box, while 30 patients were randomized into Group B, where intubation was performed without an aerosol box, as illustrated in Figure 2. One patient from Group A experienced difficult mask ventilation, necessitating the removal of the aerosol box. As a result, this case was considered a dropout. The demographic data of both groups are presented in Table 1, with no statistically significant differences observed between the groups.

**Table 1:** Demographic data Values expressed as mean + standard deviation (SD), number (percentage) or as median (interquartile range) as appropriate. Group A: With an aerosol box, Group B: Without an aerosol box ^a^Chi-squared test is used in the analysis ^b^Independent t-test is used in the analysis

	Group A with an aerosol box (n=29)	Group B without an aerosol box (n=30)	p-value
Age (years)	48.2 ± 17.8	44.9 ± 17.2	0.472^b^
Weight (kg)	62.5 ± 9.5	64.2 ± 10.4	0.511^b^
Height (cm)	162.4 ± 6.7	162.6 ± 7.7	0.950^b^
BMI (kg/m^2^)	23.4 ± 3.1	24.1 ± 3.6	0.385^b^
Gender			
Male	14 (48.3)	14 (46.7)	0.902^a^
Female	15 (51.7)	16 (53.3)	
ASA status			
I	16 (55.2)	18 (60.0)	0.708^a^
II	13 (44.8)	12 (40.0)	

**Figure 3:**
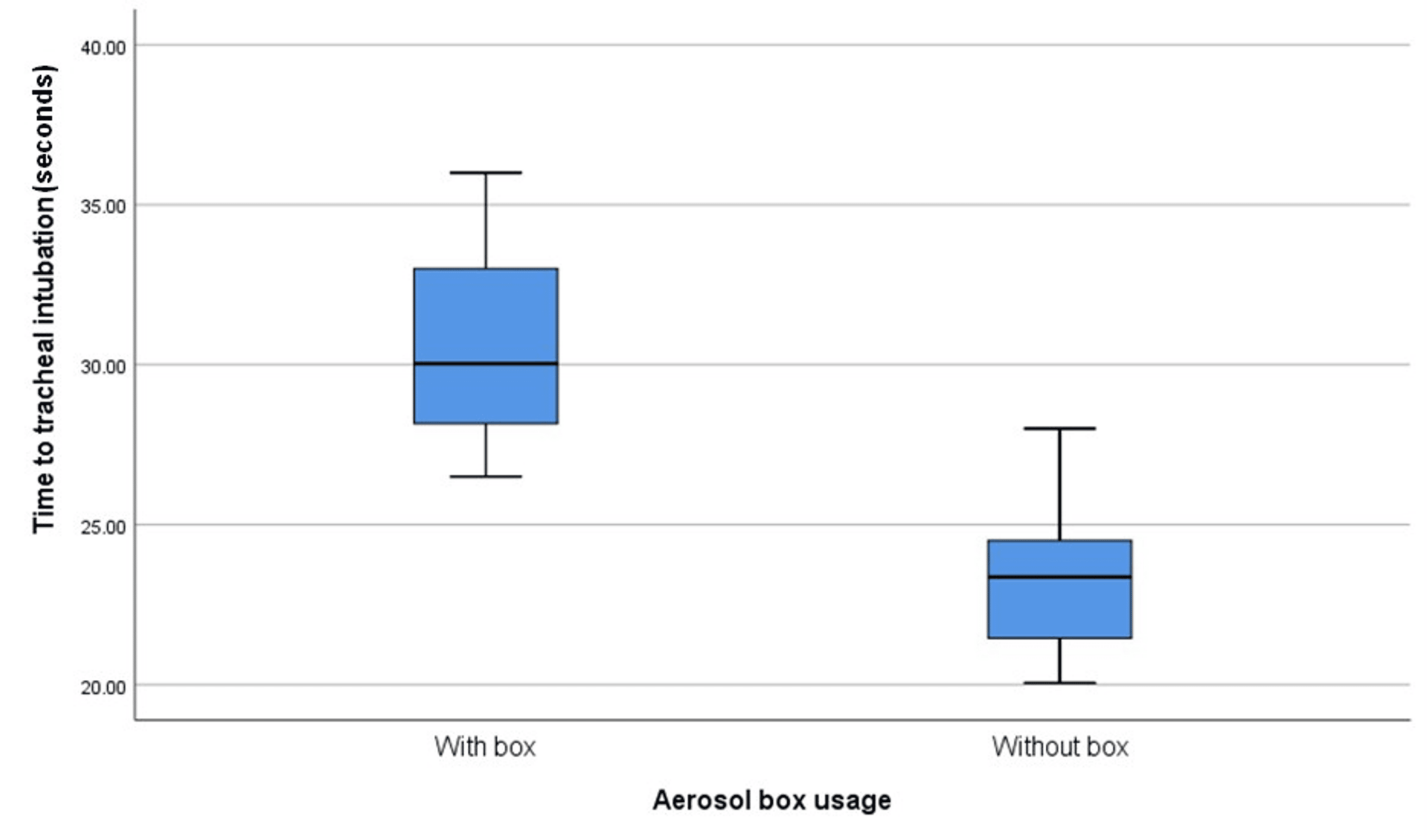
Comparison of tracheal intubation time Value expressed as mean ± standard deviation (SD). Time required for tracheal intubation using the aerosol box (mean ± SD: 30.73 ± 2.88 seconds) compared to time required for tracheal intubation without the aerosol box (mean ± SD: 23.35 ± 2.04 seconds) was significantly different using an independent t-test with p < 0.01.

All patients in the study were successfully intubated on the first attempt. Figure 3 illustrates the comparison of time to tracheal intubation in seconds between the two groups. The results demonstrated that the time required for tracheal intubation was significantly longer when performed using the aerosol box compared to intubation without the aerosol box (mean ± SD: 30.73 ± 2.88 seconds vs. 23.35 ± 2.04 seconds, p < 0.01).

No statistically significant differences were found in the association between aerosol box usage and the application of laryngeal pressure (p > 0.950), the Cormack-Lehane Grade for the first intubation attempt (p > 0.950), the intubation difficulty score (p > 0.950), or the degree of intubation difficulty (p > 0.950). Table 2 presents issues encountered by the anesthesiology trainees during the use of the aerosol box.

**Table 2:** Issues reported by the anesthesiology trainees performing tracheal intubation in the aerosol box

Issue(s)	Yes n (%)
Discomfort in arms while using the box	2 (6.9)
Laryngoscope contact with the box during intubation	6 (20.7)
Migration of the box off the bed	5 (17.2)
Assistance needed to hold the box	5 (17.2)

**Figure 4:**
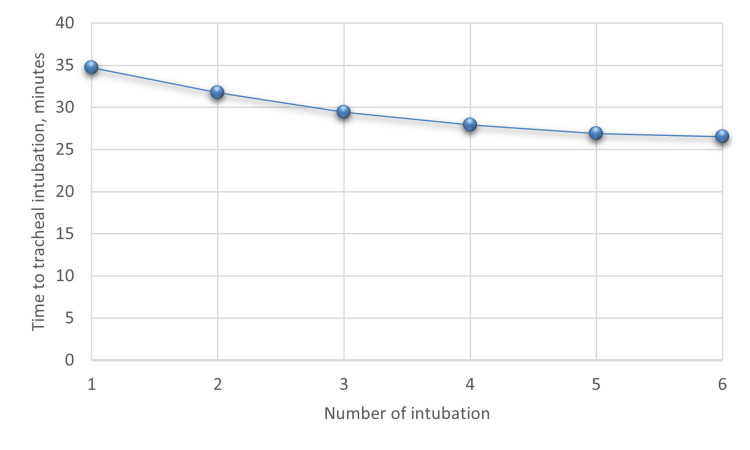
Learning curve for intubation using the aerosol box

The learning curve for intubation using the aerosol box, illustrated in Figure 4, shows a progressive reduction in intubation time as experience increased. Among the 29 patients in Group A, the mean time to tracheal intubation decreased from 32.69 seconds during the first intubation to 29.43 seconds by the third intubation and further reduced to 26.50 seconds by the sixth intubation.

## Discussion

The results of this study demonstrated that all patients in both the aerosol box and non-aerosol box groups were successfully intubated on the first attempt. However, there was a statistically significant delay in the time to tracheal intubation in the aerosol box group [8,10,11]. This finding aligns with previous studies, including an in-situ simulation crossover manikin study by Begley et al, which reported a 100% first-pass success rate for intubation without an aerosol box, compared to a 75% success rate with an aerosol box [8]. The study by Begley et al. also found that intubation time was significantly prolonged with the early-generation aerosol box compared to intubation without the box (82.1 vs. 42.9 seconds) [8]. Cheng et al. found that intubation time was significantly longer in the aerosol box group than the control group without the box, 50.3 vs 40.1 seconds [12]. Similarly, a study by de Lima et al. evaluating aerosol box use during intubation simulations reported prolonged intubation times of 25.0 seconds with the box compared to 16.0 seconds without it [6]. The delay in intubation time associated with the aerosol box was primarily attributed to the reduced ability to manipulate airway devices within the confined space. Other contributing factors included difficulty in angulating the laryngoscope and endotracheal tube inside the box [6,8]. The study by de Lima et al. further supported this finding by using accelerometers to analyze hand motions, demonstrating that the presence of an aerosol box required additional maneuvers during intubation, resulting in increased time to secure the airway [6]. 

In this study, the degree of intubation difficulty was comparable between the two groups, with no significant difficulty encountered in the aerosol box group. This outcome can be explained by the inclusion criteria, which selected patients without features of a difficult airway. Additionally, the participating anesthesiology trainees had over five years of experience in anesthesia and had undergone specific training in tracheal intubation using the aerosol box. A study by Fong et al. in 2020, which examined intubation using an aerosol box in both normal and difficult airway simulations, reported a slight increase in intubation time in normal airways (18.6 vs. 20.4 seconds) and a more pronounced delay of seven seconds (34.4 vs. 27.3 seconds) in difficult airways. Although a seven-second delay may seem minimal, it can be clinically significant in COVID-19 patients with severe respiratory impairment, as even a short delay can lead to rapid oxygen desaturation and hypoxia [10]. 

The learning curve observed in this study remained relatively consistent from the first to the sixth intubation attempt. Unlike conventional procedures, where proficiency tends to improve with repeated attempts, there was limited improvement in intubation times across participants. This finding suggests that the technical difficulties associated with using an aerosol box persist despite increasing familiarity.

Prolonged intubation time may have significant implications, particularly for vulnerable patients at risk of hypoxia, such as pediatric patients and those with severe respiratory diseases. Patients with COVID-19 pneumonia, who often have compromised respiratory function, are particularly susceptible to desaturation during airway manipulation under an aerosol box. A recent case report by Mæhlen et al. highlighted that intubation in COVID-19 patients is a high-risk procedure, where unnecessary prolongation of apnea time can lead to critical oxygen desaturation and cardiovascular collapse [13]. To date, no international guidelines, including those from the Difficult Airway Society, the Association of Anesthetists, the Intensive Care Society, the Faculty of Intensive Care Medicine, or the Royal College of Anesthetists, recommend the routine use of aerosol boxes. Similarly, Malaysia’s Guidelines on Resuscitation During the COVID-19 pandemic (Version 4/2021) recommend Level II PPE and powered air-purifying respirators for AGPs but do not endorse the use of aerosol boxes during intubation [14,15]. This lack of recommendation likely reflects safety concerns, particularly for COVID-19 patients with acute respiratory distress syndrome (ARDS), who may not tolerate even a slight delay in securing the airway. 

One patient in the aerosol box group was excluded from the study due to ineffective mask ventilation, which was likely caused by restricted arm movement, leading to suboptimal positioning for mask ventilation. A small number of participants also reported arm discomfort while using the box, unintentional contact between the laryngoscope and the box, migration of the box off the bed, and the need for an assistant to stabilize the box during intubation. In this study, the aerosol box migrated in 17.2% of tracheal intubation procedures. In contrast, Duarte-Medrano et al. reported a higher migration rate of 91.6%, likely due to the added bulk of complete PPE, which restricted arm movement [16]. This issue contributed to inefficiency, as an assistant was required to stabilize the box during intubation. Notably, these difficulties were more frequently reported by participants with larger body builds, particularly those with muscular arms or a body mass index exceeding 30, as their arm movements were further restricted within the box. A study by Azhar et al. reported similar findings, with 61% of participants experiencing limited arm mobility during intubation [17]. Additionally, these issues related to aerosol box use could be better understood and potentially addressed through a qualitative assessment, providing more detailed feedback from the trainees.

Although Azhar et al. and Cheng et al. concluded that aerosol boxes significantly reduce exposure to contamination, concerns remain regarding their use, particularly in specific patient populations. Given that multiple studies, including the present one, have consistently reported prolonged intubation times and technical challenges associated with aerosol boxes, caution is advised when considering their use in obese patients, pediatric patients, and those with ARDS [12,17-20]. 

The present study was conducted on patients to assess the impact of using an aerosol box on intubation. Therefore, it eliminated several limitations with the manikin study, as manikin anatomy is fixed and it does not mimic real physiological conditions, such as secretion and bleeding. However, this study was conducted on non-COVID-19 patients, and the participants were not donned airborne and droplet PPE. Thus, this may not mimic the real scenario of intubating COVID-19 patients. Conducting a study with the appropriate PPE would likely yield more accurate results. Additionally, blinding the trainees during tracheal intubation was not feasible due to the presence of the aerosol box.

## Conclusions

The present study demonstrated that intubation using an aerosol box achieved a 100% first-attempt success rate and did not result in significant difficulty, similar to intubation without an aerosol box. However, the use of an aerosol box significantly prolonged intubation time.
